# Macrophage activation syndrome successfully treated with eculizumab and emapalumab: a case report

**DOI:** 10.3389/fimmu.2025.1555415

**Published:** 2025-03-24

**Authors:** Paola Faggioli, Marianna Galeazzi, Carlotta Ferrari, Francesca Capelli, Chiara Marchesi, Lucia Marchionni, Laura Castelnovo, Antonio Tamburello, Eugenio Capparelli, Cristina Campidelli, Antonino Mazzone

**Affiliations:** ^1^ Department of Internal Medicine, Azienda Socio Sanitaria Territoriale (ASST) Ovest Milanese, Legnano, MI, Italy; ^2^ Department of Pathological Anatomy, Azienda Socio Sanitaria Territoriale (ASST) Ovest Milanese, Legnano, MI, Italy

**Keywords:** hemophagocytic lymphohistiocytosis (HLH), macrophage activation syndrome (MAS), eculizumab, emapalumab, thrombotic microangiopathy (TMA)

## Abstract

Hemophagocytic lymphohistiocytosis (HLH) is a hyperinflammatory syndrome, often referred to as macrophage activation syndrome (MAS) in the context of autoimmune disease-induced forms. We report the case of a 41-year-old woman with a previous diagnosis of Crohn’s disease complicated by dermatomyositis, who was admitted in our hospital for the acute onset of fever, pancytopenia, and disseminated intravascular coagulation (DIC). The laboratory findings documented hyperferritinemia, hypertransaminasemia, increased lactate-dehydrogenase (LDH), hypertriglyceridemia, and elevation of inflammatory indices, along with complement consumption. MAS was confirmed by examination of the bone marrow. Consequently, the patient was treated with high doses of glucocorticoids, subcutaneous anakinra, and intravenous immunoglobulin (IVIg). Due to the persistence of signs of thrombotic microangiopathy, we started therapy with eculizumab which stabilized the patient without improvement, so we added emapalumab, resulting in clinical improvement and normalization of blood tests.

## Introduction

Hemophagocytic lymphohistiocytosis (HLH) is a hyperinflammatory syndrome characterized by dysregulated immune activation. When HLH arises in the context of a well-defined autoimmune condition, it is more specifically referred to as macrophage activation syndrome (MAS). Both HLH and MAS are life-threatening systemic hyperinflammatory syndromes characterized by fever, elevated markers of systemic inflammation, pancytopenia, hyperferritinemia, disseminated intravascular coagulopathy, liver dysfunction, splenomegaly, and central nervous system (CNS) dysfunction (1).

Certain estimates of the prevalence and distribution of HLH within the population are difficult to establish. So far, several studies have been published on HLH incidence. Meeths M et al. published in 2015 the results from a Swedish national registry that collected data with regard to primary HLH from 1987 to 2006. They demonstrated a yearly incidence of roughly 1.5 per million (2). It is the most comprehensive data with regard to primary HLH. Interestingly, the incidence rate seems to have increased in the last years, and a recent study published by West J. et al. shows an incidence of 14.6 per million in children below 1 year, 2.2 per million in older patients (≥75 years), and a lowest incidence of 0.8 per million in those aged 15–44 years (3, 4).

There are several criteria to identify patients with HLH or MAS (5, 6). When HLH and MAS are suspected, it is essential to identify the most likely contributors, such as genetic causes, predisposing conditions, and acute triggers. HLH and MAS therapy is based on immunomodulatory drugs and on the treatment of contributing factors.

## Case description

Here we describe the case of a 41-year-old woman suffering from Crohn’s disease complicated by dermatomyositis. The patient was diagnosed with Crohn’s disease, predominantly involving the ileum at the age of 16, and she was initially treated with mesalazine and prednison. At the age of 20, mesalazine was discontinued following a concurrent diagnosis of dermatomyositis, which was subsequently managed with azathioprine, cyclosporine, and methotrexate. However, the new treatments proved ineffective, and prednisone was reintroduced. At the age of 41, in September and October 2023, the patient was prescribed her first biological disease-modifying antirheumatic drug (DMARD), infliximab, at a dose of 5 mg/kg. The 300-mg drug was administered twice, resulting in the successful management of Crohn’s disease.

In December 2023, she was hospitalized for 10 days long due to a persistent fever despite the use of azithromycin and cefixime. No other symptoms were present.

On examination, her temperature was 38.6°C, heart rate was 110 beats/minute with regular rhythm, blood pressure was 110/70 mmHg, and oxygen saturation was 96%. The Glasgow Coma Scale score was 15. The cardiovascular and pulmonary examinations were normal. We found hepatomegaly at liver palpation and ecchymosis on the limbs. The blood tests documented the following results: white blood cell count of 0.9 × 10^3^/μL (normal range, 4–10 × 10^3^/μL), hemoglobin level of 79 g/L (normal range, 120–160 g/L), and platelet count of 39 × 10^3^/μL (normal range, 150–440 × 10^3^/μL). The sodium level was 129 mmol/l (normal range, 135–145 mmol/L). The other serum electrolyte and renal function tests had results in the normal range. Proteinuria was absent. The liver function tests showed an aspartate aminotransferase level of 476 U/L (normal range, ≤40 U/L), an alanine aminotransferase level of 175 U/L (normal range, ≤45 U/L), a lactate dehydrogenase level of 1,800 U/L (normal range, 135–214 U/L), a ferritin level of 9,308 ng/mL (normal range, 10–150 ng/mL), a D-dimer level of 27,662 ng/mL (normal range, ≤270 ng/mL), a fibrinogen level of 39 mg/dL (normal range, 180–400 mg/dL), a serum level of triglycerides of 419 mg/dL (normal range, ≤200 mg/dL), a C-reactive protein level of 6.30 mg/dL (normal range, ≤0.5 mg/dL), a complement component C3 level of 53 mg/dL (normal range, 90–180 mg/dL), and a complement component C4 level of 8 mg/dL (normal range, 10–40 mg/dL).

The blood and urine cultures were negative. The chest X-ray showed normal findings. The test for detection of SARS-CoV2 was negative.

To investigate possible septic foci, the patient underwent a computer tomography (CT) of the chest and abdomen. The CT showed ground glass opacity in the lungs (in particular, bilaterally in the lung bases) and inhomogeneity of the rectus abdominal muscles.

**Figure 1:**
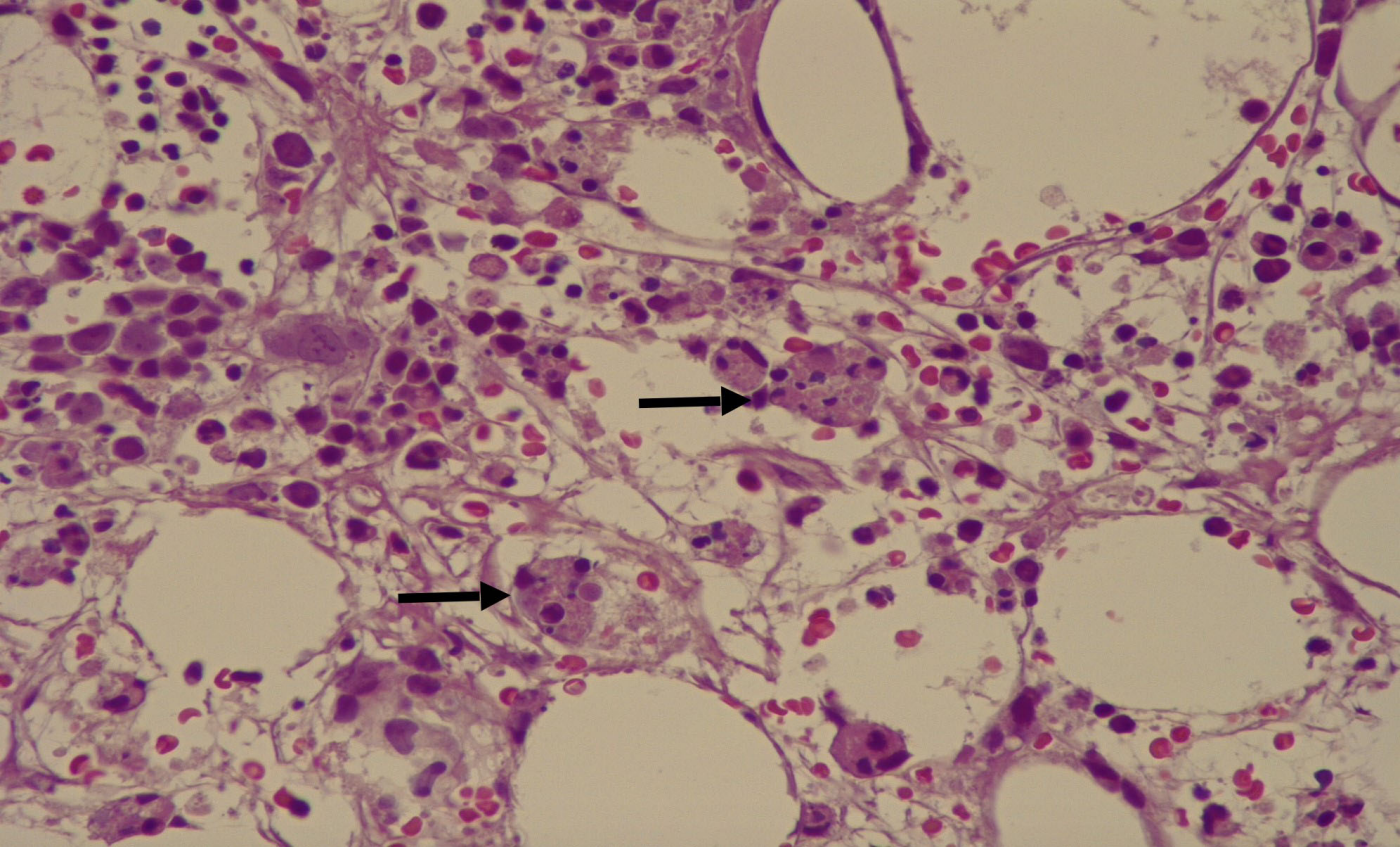
Bone marrow biopsy with macrophages (CD68+) containing numerous red blood cells in their cytoplasm (arrow).

Furthermore, we documented possibly recent Epstein–Barr virus (EBV) and *Mycoplasma pneumoniae* infections due to the presence of anti-VCA IgG, EBNA antibodies IgG, and anti-*Mycoplasma pneumoniae* IgM. EBV DNA was undetectable. The patient received antibiotic therapy with cephalosporin (piperacillin/tazobactam) and teicoplanin as prescibed by infectivologists. Initially, the clinical presentation could subtend a sepsis. A rheumatology evaluation on the admission day concluded for MAS (laboratory tests met the criteria: fever, elevated level of C-reactive protein, elevated level of lactate dehydrogenase, pancytopenia, hyperferritinemia, elevated level of aspartate aminotransferase and alanine aminotransferase, and elevated serum level of triglycerides), and disseminated intravascular coagulation in patients with suspected infections that could contribute to the MAS relapse (5, 6). MAS was confirmed by examination of the bone marrow (**Figure 1**).

An initial treatment with a high dose of glucocorticoids (methylprednisolone, 2 mg/kg daily), subcutaneous anti-IL1R antagonist anakinra (1,200 mg/day), and intravenous immunoglobulins (IVIg) at a dosage of 1 g/kg/day for two consecutive days was established.

For fever relapse, the antibiotic therapy was changed: piperacillin/tazobactam was discontinued, and meropenem, levofloxacin, and caspofungin were added as prescribed by infectivologists.

Due to the persistence of fever and signs of thrombotic microangiopathy (TMA), in particular, hypocomplementemia, thrombocytopenia, anemia, red blood cell fragmentation (schistocytes) on peripheral blood smear, and fever, an add-on therapy with eculizumab was prescribed. We reached stabilization but no improvement so that the patient was consequently switched to emapalumab. Emapalumab, an anti-IFNγ monoclonal antibody, required the following dosages: 6 mg/kg on the first day, 3 mg/kg every 3 days until the 15th day, and 3 mg/kg twice a week until the 28th day. The therapy should be stopped at disease remission but not before three administrations have taken place. Emapalumab was subsequently discontinued due to clinical improvement and normalization of LDH, complement levels, and fibrinogen.

**Figure 2:**
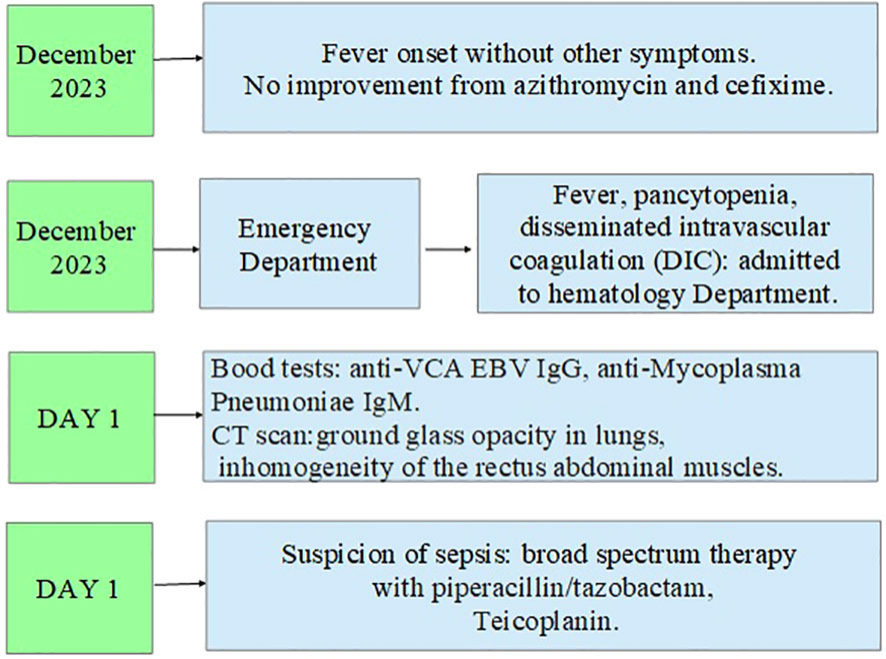
First part of the clinical course.

**Figure 3:**
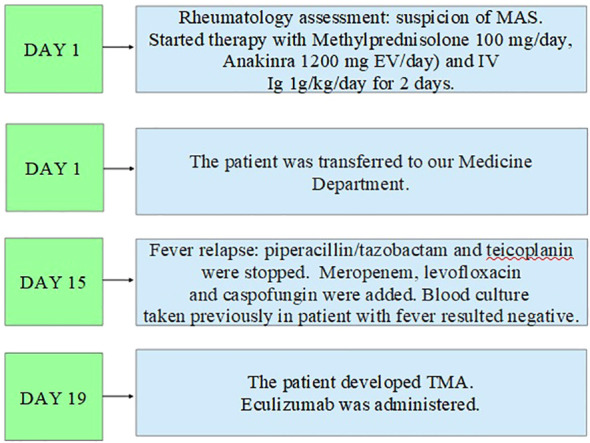
Second part of the clinical course.

**Figure 4:**
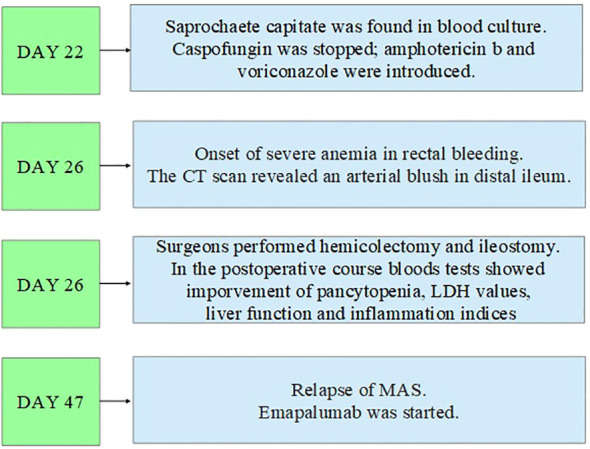
Third part of the clinical course.

In addition, the clinical course was complicated by *Saprochaete capitata* sepsis that required treatment with amphotericin b and voriconazole. Moreover, the patient also experienced an episode of severe rectal bleeding that needed surgery intervention with hemicolectomy and subsequent ileostomy. In **Figures 2**–**4**, a flow chart is reported in order to summarize the clinical course.

At discharge, the patient was asymptomatic, the laboratory tests were normal as reported in **Table 1**, and the therapy included prednisone at a dosage of 50 mg per day, with plans for gradual tapering.

**Table 1:** Laboratory tests at admission, after 14 days of treatment with anakinra and steroids, at development of TMA (day 19), at the end of eculizumab treatment, at initiation of emapalumab (day 47), and at discharge.

Laboratory tests	Results—day 1	Results—day 15 (14 days of anakinra and steroid treatment)	Results—day 19: TMA development and initiation of eculizumab	Results—day 47: initiation of emapalumab	Results—discharge day	Reference range
White blood cell count, ×10^9^/L	0.9	4.4	0.8	7.1	4.3	4–10 × 10^9^/L
Neutrophils, ×10^9^/L	0.6	0.5	0.3	4.5	2.6	2–7 × 10^9^/L
Hemoglobin, g/dL	76	86	47	92	95	120–160 g/L
Platelets, ×10^9^/L	39	236	48	206	223	150–440 × 10^9^/L
Aspartate aminotransferase, U/L	476	139	168	42	13	≤40 U/L
Alanine aminotransferase, U/L	175	29	30	56	30	≤45 U/L
Lactate dehydrogenase, U/L	>1,800	1,265	>1,800	240	132	135–214 U/L
Ferritin, ng/mL	9,308	17,473	32,059	595	325	10–150 ng/mL
D-dimer, ng/mL	27,662	39,138	9,429	6,333	604	≤270 ng/mL
Complement component C3, mg/dL	53	96	44	52	118	90–180 mg/dL
Complement component C4, mg/dL	8	9	8	5	18	10–40 mg/dL
Fibrinogen, mg/dL	39	88	58	94	197	10–150 ng/mL

The patient felt a sense of relief at discharge. During hospitalization, she experienced moments of deep concern for her situation.

At the last follow-up in September 2024, the patient was maintained on prednisone at a reduced dose of 10 mg per day, which corresponds to the typical maintenance dose for CD. The patient is currently in clinical remission and is being evaluated for potential stoma reversal surgery. The laboratory tests indicated significant improvements, with CRP and erythrocyte sedimentation rate (ESR) levels within normal limits and complement components and hematological parameters restituted to normal values.

## Discussion

At first, we started to treat MAS using glucocorticoids (methylprednisolone at 100 mg per day), anakinra (1,200 mg per day), and IVIg (1 g/kg for 2 days) as defined by the guidelines (1). We also treated the underlying disease with cephalosporin (piperacillin/tazobactam), teicoplanin, and antifungals as recommended (7).

Unfortunately, the patient developed worsening TMA. The laboratory tests showed lower levels of hemoglobin, platelet count, and fibrinogen and a higher level of lactate dehydrogenase and D-dimer. Hypocomplementemia persisted as concurrent clinical and serological findings in the context of MAS.

As regard to the latter, in 2011, Gorelik M et al. described hypocomplementemia associated with MAS in two patients with systemic juvenile idiopathic arthritis (sJIA) and in one patient with adult-onset Still’s disease (AOSD) (8). More recently, some authors have suggested that the control of the complement pathway may lead to the successful control of complement-mediated TMA in the context of MAS (9, 10).

The patient did not present kidney and central nervous system involvement as TMA complication, but she developed rectal bleeding that needed surgery. The surgeons performed hemicolectomy and ileostomy.

To investigate other potential causes of TMA, we measured the activity of ADAMTS13, a von Willebrand factor-cleaving protease that cuts von Willebrand factor multimers secreted from vascular endothelial cells. ADAMTS13 activity was 93% (normal range 61–131). The result could rule out a diagnosis of thrombotic thrombocytopenic purpura.

The association between TMA and MAS has rarely been assessed, but it is reported with increased frequency (9–11). The coexistence of TMA and MAS has been outlined as a complication of hematopoietic stem cell transplantation (12, 13), renal transplantation (14), autoimmune disease in children (9), and complement-mediated TMA (15) and, recently, associated to systemic lupus erythematosus (SLE) (11).

In the study “Thrombotic Microangiopathy Associated with Macrophage Activation Syndrome: A Multinational Study of 23 Patients”, the authors reported the therapies used for MAS (cyclosporine, anakinra, etoposide, and intravenous immunoglobulins) and for TMA (plasma exchange, eculizumab, rituximab, cyclophosphamide, and mycophenolate) (9).

In 2023, Yamaguchi M et al. published a case report of TMA concomitant with MAS in systemic lupus erythematosus, refractory to conventional treatment, and successfully treated with eculizumab. In the two abovementioned studies, emapalumab was not used (11).

In 2020, Gloude NJ et al. published data regarding 16 patients with HLH and TMA treated with emapalumab and eculizumab. They hypothesized that high levels of interferon gamma in HLH might contribute directly to endothelial damage or injure the endothelium through complement system activation (10). Considering that also in systemic juvenile idiopathic arthritis-associated MAS a prominent pathogenetic role of interferon gamma has been demonstrated, the same authors suggested that the pathophysiology of TMA in MAS is similar to the one hypothesized for the other forms of HLH (10). Our case report could confirm their hypothesis.

The patient underwent eculizumab treatment at 900 mg/week for 4 weeks as suggested by the guidelines (16). The patient also received blood product or component transfusion. After 4 weeks, we obtained stabilization without improvement. In particular, the blood tests showed a low level of fibrinogen and a higher level of D-dimer, while the white blood cell count and hemoglobin levels rose without reaching normal values.

In the light of MAS relapse, the patient was treated with emapalumab, with clinical improvement and normalization of LDH, complement, and fibrinogen. Emapalumab is the first targeted therapy approved by the US FDA for primary pediatric HLH (17). Emapalumab is a monoclonal antibody that binds and neutralizes interferon gamma. It binds free and receptor-bound interferon gamma and neutralizes its biologic activity, reducing hyperinflammation.

The choice of emapalumab rather than etoposide or JAK2 inhibitors stems from several reasons. First of all, as mentioned earlier, emapalumab is the first targeted therapy approved by the US FDA for primary pediatric HLH. Secondly, the absence of EBV DNA and underlying hematological malignancies allowed us to rule out etoposide (1). Thirdly, with regard to the use of JAK2 inhibitors, there are a few reports in literature of patients receiving JAKi treatment (18).

Furthermore, it has been demonstrated that patients affected by TMA and HLH who received the complement blocker eculizumab in addition to the interferon gamma inhibitor emapalumab had complete resolution of TMA and HLH (10).

## Conclusions

Ours is one of the first reports of the use of emapalumab and eculizumab in adult patients with MAS and TMA: the drug emapalumab has proven to be effective in adults for a pathology burdened with a very high mortality rate.

## Data Availability

The original contributions presented in the study are included in the article/supplementary material. Further inquiries can be directed to the corresponding authors.
